# Socioeconomic Status and Childhood Leukemia Incidence in Switzerland

**DOI:** 10.3389/fonc.2015.00139

**Published:** 2015-06-30

**Authors:** Martin Adam, Claudia E. Kuehni, Adrian Spoerri, Kurt Schmidlin, Fabienne Gumy-Pause, Pierluigi Brazzola, Nicole Probst-Hensch, Marcel Zwahlen

**Affiliations:** ^1^Swiss Tropical and Public Health Institute, Basel, Switzerland; ^2^University of Basel, Basel, Switzerland; ^3^Institute of Social and Preventive Medicine, University of Bern, Bern, Switzerland; ^4^Haematology/Oncology Unit, Department of Paediatrics, University Hospital of Geneva, Geneva, Switzerland; ^5^Department of Paediatrics, Ospedale San Giovanni, Bellinzona, Switzerland

**Keywords:** childhood cancer, leukemia, socioeconomic status, risk factor, case–control study

## Abstract

Socioeconomic status (SES) discrepancies exist for child and adult cancer morbidity and are a major public health concern. In this Swiss population-based matched case–control study on the etiology of childhood leukemia, we selected the cases from the Swiss Childhood Cancer Registry diagnosed since 1991 and the controls randomly from census. We assigned eight controls per case from the 1990 and 2000 census and matched them by the year of birth and gender. SES information for both cases and controls was obtained from census records by probabilistic record linkage. We investigated the association of SES with childhood leukemia in Switzerland, and explored whether it varied with different definitions of socioeconomic status (parental education, living condition, area-based SES), time period, and age. In conditional logistic regression analyses of 565 leukemia cases and 4433 controls, we found no consistent evidence for an association between SES and childhood leukemia. The odds ratio comparing the highest with the lowest SES category ranged from 0.95 (95% CI: 0.71–1.26; *P*_trend_ = 0.73) for paternal education to 1.37 (1.00–1.89; *P*_trend_ = 0.064) for maternal education. No effect modification was found for time period and age at diagnosis. Based on this population-based study, which avoided participation and reporting bias, we assume the potential association of socioeconomic status and childhood leukemia if existing to be small. This study did not find evidence that socioeconomic status, of Switzerland or comparable countries, is a relevant risk factor or strong confounder in etiological investigations on childhood leukemia.

## Introduction

In Switzerland and other western countries, childhood cancer is the second leading cause of death in children (1, 2). About 200 new diagnoses of cancer in children younger than 15 years are annually registered in the Swiss Childhood Cancer Registry (SCCR) with leukemia accounting for about one-third of all diagnoses (3–5). Incidence numbers all over Europe show some evidence for an increase of leukemia during the past decades (6, 7). Risk factors for childhood leukemia are poorly understood, most likely involving the interplay of environmental and genetic factors (8–12). Inequalities in health between socioeconomic groups are a major public health concern. Epidemiological studies found higher rates of all-cause mortality and morbidity among infants, children, and adults of lower socioeconomic status (SES), defined at an individual or area-level (13–15). Childhood leukemia and similarly acute lymphoblastic leukemia (ALL), the most common subtype, have been reported to be one of the rare exceptions, being more common among children of high SES (16–18). This led to speculations about a large range of potential etiological factors linked with affluence and modern lifestyle, which could act in part via altered host susceptibility (16–18). Two reviews, summarizing the evidence on SES and childhood leukemia until August 2002 (19) and April 2008 (20), concluded that the results of these studies were heterogeneous and varied by place, time, study design, leukemia subgroup, age at diagnosis, and measures of SES used. They advised future studies to minimize bias in selecting cases and controls, to distinguish between different SES measures, and between leukemia subtypes.

In Switzerland, the existence of the population-based SCCR (5, 21) and the Swiss National Cohort (SNC) (5, 22) provided an ideal opportunity to study these questions. We linked childhood leukemia cases from SCCR to SNC and conducted a matched case–control study to investigate the association between socioeconomic status and incidence of childhood leukemia. We explored whether this association varied with different definitions of SES (parental education, living condition, area-based SES), with time period and with age at diagnosis.

## Materials and Methods

### Study population and data sources

The Swiss Childhood Cancer Registry (SCCR)^1^ started in 1976 to register all patients treated in one of the nine pediatric cancer centers in Switzerland located in the tertiary care pediatric hospitals in Aarau, Basel, Bern, Geneva, Lausanne, Locarno, Lucerne, St. Gallen, and Zurich (5, 21). Physicians treating these children are members of the Swiss Paediatric Oncology Group (SPOG). The registry includes children and adolescents up to the age of 20 years and aims to be complete for Switzerland in those aged 0–15 years as this age range should be treated in pediatric cancer centers (23). The database contains clinical information on past medical history, cancer diagnosis, treatment, follow-up, as well as cause and date of death. The Swiss National Cohort (SNC)^2^ is a long-term, census-based, cohort study of the Swiss-resident population (22). It is based on individual data from the census 1990 and the census 2000 in Switzerland. Mortality records from 1991 up to 2008 have been linked to this cohort using probabilistic record linkage procedures.

**Figure 1:**
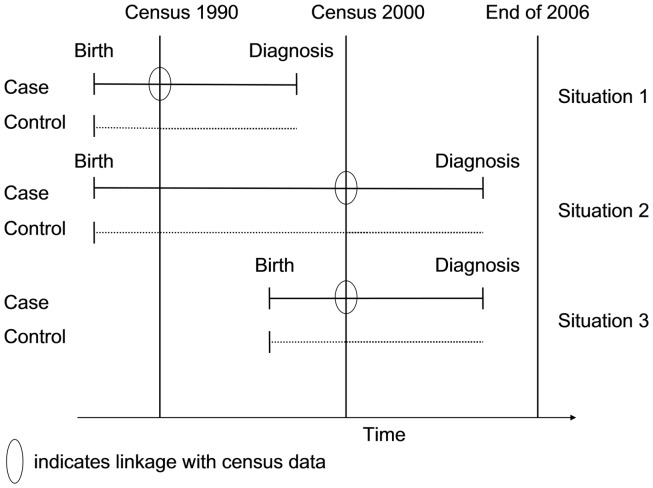
**Schematic description of the included cases and controls and the linkage with census information**. Only childhood cancer cases born before a census and diagnosed after a census were eligible to be included as cases; controls were matched for year of birth and gender.

To investigate the association between socioeconomic status and risk of leukemia in Switzerland, we used a case–control study design. Data on cases, with the exception of the SES information, was obtained through the SCCR. Controls were selected from the census 1990 and 2000. Using a probabilistic linkage procedure, cases were linked to the census 1990 and census 2000 datasets, to obtain SES information for the cases (24). By design, we wanted SES information to be available from before the date of diagnosis. Cases therefore needed to be born before one of the censuses (1990 or 2000), and the disease had to be diagnosed after the census (Figure 1). This circumvented the problem of including cases with a diagnosis before the census that had died prior to the census. Subject to this restriction, the study included as cases all children in the SCCR who were resident in Switzerland, aged <16 years at diagnosis (the age group for which the registry achieves highest coverage) and had been diagnosed with leukemia [Diagnostic group I of the International Classification of Childhood Cancer, third revision (ICCC3)] (25). For the probabilistic linkage with the census, the childhood cancer patients from the SCCR were divided into two subgroups. All cases born before census 1990 and diagnosed between January 1991 and December 2000 were assigned to the “census 1990 case subset” (situation 1 in Figure 1); all cases born before census 2000 and diagnosed between January 2001 and December 2006 (reported to the SCCR by December 2007 when this study was initiated) were assigned to the “census 2000 case subset” (situations 2 and 3 in Figure 1). For every case, we randomly selected eight control children from the two census rounds (census 1990 and census 2000) and matched them individually to the cases by gender and birth year. Our study was designed as a case cohort study (26, 27). We did not attempt to exclude diseased children from the control set, as the theoretical foundation of case–control studies allows to include as controls a representative sample from the source population from which the cases arise, thus providing an unbiased estimate of the exposure–disease risk ratio (26).

### Linkage with the swiss national cohort

The probabilistic record linkage was done based on variables available both in the census records and in the SCCR: sex, date of birth, place of residence (Data Sheet 1 in Supplementary Material). In some instances, several possible matches per records with different probability weights were found and we therefore prepared three different data sets for sensitivity analyses: (1) best links data set, (2) second best links data set, and (3) third best links data set.

### Assignment of socioeconomic status and operational definitions of SES

To assign a child meaningful SES information, the child had to live in a family household. We restricted our study to cases and controls living in a family household for which a reference adult person could be assigned by the Swiss Federal Statistical Office. The reference person of a household (i.e., the householder) being responsible for the socioeconomic position of the household is assigned in the census according to the following criteria: job position, role in the household, age (named “mother” if female reference person or partner of the reference person, respectively “father” if male). Based on previous publications, which had shown conflicting results with varying SES definitions, we had decided to use several variables available in the census as indicators of SES, including individual-based SES information (education of the mother or the father in the household), household-based information (number of rooms per person excluding kitchen and bathrooms, square meter living space per person), and a publicly available area-based SES index (SES index) as developed by the Department of Geography at the University of Zurich^3^. The area-based SES index is constructed to reflect the social status of the population-based information about net income, education level, and job position in the respective area (community or quarter) (28). We created categories for the SES information as follows. We created three levels for the living space per person and for the area-based SES index based on tertiles with lower values representing the lower SES and the higher values higher SES. For additional analyses, we also created five levels based on quintiles. Parental education was grouped into three categories: “compulsory schooling or less” (up to 9 years of education), “secondary education” (10–16 years, high school, teachers training colleges, technical colleges, and upper vocational education), and “tertiary education” (16 years or more). Number of habitable rooms per person was grouped into three categories based on cut-offs, which can easily be interpreted: <1 room per person [=more than 1 person per room (ppr >1 = overcrowded), 1–1.25 room per person, >1.25 room per person (29)].

### Statistical analyses

We described the characteristics of the children and their parents for leukemia cases and controls. We fitted conditional logistic regressions models that account for the matched case–control study design, to assess the association of SES with the odds of having leukemia. We report odds ratios with 95% confidence intervals and statistical significance based on Wald tests. To account for possible confounding factors, we fitted multivariable conditional logistic regression models adjusting for mother’s age, father’s age, nationality, language region, and number of older children in household. To calculate a *P*-value for trend over the SES levels, we included SES as a continuous variable coded from 1 to 3. In additional analysis, incorporating appropriately constructed terms for effect modification in the conditional logistic regression models, we examined whether the association with SES measures differed by time period (census 1990 versus census 2000) or by age at diagnosis (0–4 years versus 5–15 years). All the analyses were repeated including only acute lymphoblastic leukemia (ALL) cases and their matched controls. Because it had been suggested that associations with SES might only be seen in the most extreme groups (highest or lowest 10–20%) (16, 20, 30, 31), we also performed an analysis comparing only the highest 20% (highest quintile) to the lowest 20% (lowest quintile) for the SES measures living space and area-based SES index. Finally, we performed a sensitivity analysis with regard to linkage probability, by repeating the conditional logistic regressions for the second best links and third best links datasets (Tables S1 and S2 in Supplementary Material).

All *P*-values are two sided and a *P*-value ≤0.05 indicated statistical significance. All statistical analyses were performed using STATA, version 10 (StataCorp., 2005. Stata Statistical Software: Release 10.1. College Station, TX, USA: StataCorp LP).

### Consent and approval

The SCCR has national approval to collect information on children with childhood cancer. Parents are granted the right to demand that the data on their children in the SCCR is anonymized. The data of the Swiss National Cohort are fully anonymous, and approval for the design and conduct of the SES project was given by the Swiss Federal Statistical Office via a specific legal contract. The project data are fully anonymous and kept separate from both SNC and SCCR. By design, this purely observational and anonymous study could not obtain individual informed consent.

## Results

### Demographic and clinical characteristics of the patients

Almost 100% (565 of 566) of leukemia cases registered in the SCCR and meeting the inclusion criteria of this study could be linked to the census 1990 or to the census 2000. To 559 of 565 leukemia cases (98.9%) and to 4433 of the 4520 controls (98.1%), a householder could be assigned to the household of the child by the Swiss Federal Statistical Office. Therefore, the leukemia case–control study file for analyzing the association with SES levels consisted of 559 cases and 4433 matched controls with an average control to case ratio of 7.9 (in some instances, <8 matching controls were available). Of the 559 leukemia patients, 425 (76%) were diagnosed with an acute lymphoblastic leukemia (ALL) and 60% (334/559) were male (Table 1). Roughly 28% of all leukemia patients and about 32% of the ALL patients were younger than 5 years of age at time of diagnosis. The distortion of the usual age distribution of leukemia/ALL is explained by the linkage design (born before census, diagnosed after census), giving older children a higher likelihood of inclusion into the study. At the time of birth, the mother was on average 29.2 (±5.3 years) years of age and the father 32.3 (±6.0). Half of the cases had older children in the household. Similar to the distribution in the general population, 78.4% (438/559) leukemia cases were Swiss and 76.4% (427/559) were from the German speaking part of Switzerland.

**Table 1:** **Demographic and clinical characteristics of leukemia (and ALL) patients**.

Characteristics	Leukemia				ALL			
	Cases		Controls		Cases		Controls	
	*N*	%	*N*	%	*N*	%	*N*	%
**Total**	559	100	4433	100	425	100	3350	100
**Sex of case/control child**								
Male	334	59.7	2640	59.6	250	58.8	1961	58.5
Female	225	40.3	1793	40.4	175	41.2	1389	41.5
**Age at diagnosis (in years) of case child**								
0–4	156	27.9			135	31.8		
5–9	188	33.6			145	34.1		
10–13	150	26.8			107	25.2		
14–15	65	11.6			38	8.9		
**Age at census (in years) of case/control child**								
<1	76	13.6	616	13.9	60	14.1	487	14.5
1–4	234	41.9	1848	41.7	193	45.4	1511	45.1
5–9	167	29.9	1327	29.9	119	28.0	944	28.2
10–13	75	13.4	592	13.4	48	11.3	373	11.1
14–15	7	1.3	50	1.1	5	1.2	35	1.0
**Age of the mother at child birth (in years)**								
<25	106	19.0	893	20.3	70	16.5	672	20.2
25–29	203	36.4	1732	39.3	154	36.4	1308	39.3
30–34	174	31.2	1263	28.7	135	31.9	950	28.6
35+	74	13.3	514	11.7	64	15.1	396	11.9
Missing	2		31		2		24	
**Age of the father at child birth (in years)**								
<25	50	9.5	331	7.9	32	8.0	234	7.4
25–29	136	25.8	1283	30.7	100	25.0	976	30.8
30–34	185	35.1	1487	35.6	138	34.5	1125	35.5
35+	156	29.6	1075	25.7	130	32.5	831	26.2
Missing	32		257		25		184	
**Total number of children in household**								
1	144	25.8	1099	24.8	109	25.6	850	25.4
2	268	47.9	2137	48.2	205	48.2	1599	47.7
3	102	18.2	851	19.2	77	18.1	642	19.2
4+	45	8.1	346	7.8	34	8.0	259	7.7
**Number of children in household who are older than case or control**								
0	275	49.2	2179	49.2	206	48.5	1632	48.7
1	206	36.9	1631	36.8	158	37.2	1221	36.4
2+	78	14.0	623	14.1	61	14.4	497	14.8
**Nationality**								
Swiss	438	78.4	3481	78.5	341	80.2	2629	78.5
Non-Swiss	121	21.6	952	21.5	84	19.8	721	21.5
**Language region**								
German	427	76.4	3237	73.0	324	76.2	2447	73.0
French	112	20.0	1034	23.3	90	21.2	794	23.7
Italian	20	3.6	162	3.7	11	2.6	109	3.3
**Census**								
1990	300	53.7	2376	53.6	218	51.3	1724	51.5
2000	259	46.3	2057	46.4	207	48.7	1626	48.5

*ALL indicates acute lymphoblastic leukemia*.

### Socioeconomic characteristics of the patients

The distribution according to the available SES information for cases and controls is given in Table 2. Over 90% (504/559) of the assigned householders were males and married. In 42% of the households (234/559), both father and mother were working, and in 55% of the households (310/559) only one of the parents worked. The large majority [97.2% (512/527)] of the fathers was employed and 78.9% (413/527) had at least a secondary education. Of the mothers, 47.8% (266/557) were employed and 71.7% (399/557) had at least a secondary education. About 27% (149/559) of the households of cases had <1 room per person and a third had <23 m^2^ living space per person.

**Table 2:** **Socioeconomic characteristics of leukemia (and ALL) patients**.

Characteristics	Leukemia				ALL			
	Cases		Controls		Cases		Controls	
	*N*	%	*N*	%	*N*	%	*N*	%
**Total**	559		4433		425		3350	
**Employment status of the mother**								
Employed	266	47.8	2104	47.8	198	46.8	1593	47.9
Not employed	291	52.2	2298	52.2	225	53.2	1733	52.1
Missing	2		31		2		24	
**Employment status of the father**								
Employed	512	97.2	4045	96.9	388	97.0	3067	96.9
Not employed	15	2.8	131	3.1	12	3.0	99	3.1
Missing	32		257		25		184	
**Education status of the mother**								
Compulsory education	158	28.4	1365	31.0	119	28.1	1032	31.0
Secondary education	324	58.2	2538	57.7	248	58.6	1916	57.6
Tertiary education	75	13.5	499	11.3	56	13.2	378	11.4
Missing	2		31		2		24	
**Education status of the father**								
Compulsory education	114	21.6	879	21.0	86	21.5	671	21.2
Secondary education	258	49.0	2061	49.4	195	48.8	1546	48.8
Tertiary education	155	29.4	1236	29.6	119	29.8	949	30.0
Missing	32		257		25		184	
**Rooms per person**								
<1 room/person	149	26.9	1168	26.6	110	26.3	886	26.7
1–1.25 room/person	206	37.3	1631	37.1	162	38.7	1234	37.2
>1.25 room/person	198	35.8	1593	36.3	147	35.1	1197	36.1
Missing	6		41		6		33	

*ALL indicates acute lymphoblastic leukemia*.

### Association between SES measures and childhood leukemia

We used conditional logistic regression analyses to estimate the strength of the association between various SES measures and the risk of childhood leukemia (Table 3), analyzing all leukemia patients (left columns) or ALL patients only (right columns). Results were very similar for the unadjusted analysis, including only the SES characteristic, and for the adjusted analysis additionally including mother’s age, father’s age, nationality, language region, and older children in household as additional exposures. Analyses using the individual-based (education of the mother, education of the father), the household-based SES measures (rooms per person and square meter of living space per person), and the area-based SES index showed no consistent association. Only for maternal education, there was a weak trend for an increased risk of leukemia in children of mothers with tertiary education compared to mothers with compulsory schooling (OR1.37, 95% CI 1.00–1.89; *P*_trend_ = 0.064). This association was less pronounced for ALL cases (OR 1.19, 95% CI 0.82–1.73; *P*_trend_ = 0.39). For a higher educational level of the father (OR 0.95, 95% CI 0.71–1.26; *P*_trend_ = 0.73) and square meter per person (OR 0.96, 95% CI 0.74–1.25; *P*_trend_ = 0.78), the risk of childhood leukemia was slightly decreased in the highest SES group. Both, living space in square meter (OR 1.18, 95% CI 0.90–1.54; *P*_trend_ = 0.22) and the area-based SES index (OR 1.21, 95% CI 0.94–1.55; *P*_trend_ = 0.34), showed an increased leukemia risk of about a factor 1.2 for the upper SES tertile group compared to the lower tertile group but showing no clear incremental trend. Comparing highest with lowest 20% (instead of tertiles) for living space and for area-based SES index, the ORs for the highest 20% showed weak elevated risks with imprecise confidence intervals.

**Table 3:** **Risk association (odds ratio and 95% confidence intervals) of socioeconomic status with leukemia (and ALL), by type of SES characteristic**.

SES	Univariable analysis										Multivariable analysis^a^									
	Leukemia					ALL					Leukemia					ALL				
	*N*	OR	(95%CI)	*P*	*P*_trend_	*N*	OR	(95%CI)	*P*	*P*_trend_	*N*	OR	(95%CI)	*P*	*P*_trend_	*N*	OR	(95%CI)	*P*	*P*_trend_
**Education status of the mother**	4897					3710					4387					3330				
Compulsory education		1					1					1					1			
Secondary education		1.10	(0.90–1.35)	0.345	0.084		1.12	(0.89–1.41)	0.329	0.13		1.11	(0.88–1.40)	0.385	0.064		1.05	(0.80–1.36)	0.733	0.39
Tertiary education		1.30	(0.97–1.75)	0.076			1.30	(0.92–1.82)	0.134			1.37	(1.00–1.89)	0.052			1.19	(0.82–1.73)	0.353	
**Education status of the father**	4427					3365					4387					3330				
Compulsory education		1					1					1					1			
Secondary education		0.96	(0.76–1.22)	0.751	0.81		0.99	(0.75–1.30)	0.938	0.87		0.96	(0.73–1.26)	0.770	0.73		0.92	(0.68–1.25)	0.586	0.38
Tertiary education		0.97	(0.75–1.25)	0.793			0.98	(0.73–1.31)	0.873			0.95	(0.71–1.26)	0.712			0.86	(0.62–1.20)	0.382	
**Rooms per person**	4856					3670					4313					3261				
<1 room/person		1					1					1					1			
1–1.25 room/person		0.99	(0.79–1.24)	0.946	0.80		1.06	(0.82–1.37)	0.673	0.88		0.98	(0.77–1.24)	0.860	0.78		1.00	(0.76–1.32)	0.982	0.50
>1.25 room/person		0.97	(0.77–1.22)	0.808			0.99	(0.76–1.28)	0.918			0.96	(0.74–1.25)	0.775			0.91	(0.67–1.23)	0.525	
**Living space (in m^2^)**	3643					2733					3246					2438				
Lower tertile		1					1					1					1			
Medium tertile		0.93	(0.73–1.18)	0.537	0.25		0.90	(0.68–1.18)	0.430	0.39		0.97	(0.75–1.25)	0.795	0.22		0.90	(0.67–1.21)	0.476	0.62
Upper tertile		1.15	(0.91–1.46)	0.236			1.13	(0.86–1.48)	0.375			1.18	(0.90–1.54)	0.220			1.08	(0.80–1.47)	0.621	
Lowest 20%		1					1					1.00					1			
Highest 20%		1.18	(0.87–1.59)	0.288			1.26	(0.89–1.78)	0.196			1.27	(0.90–1.80)	0.167			1.26	(0.85–1.86)	0.257	
**Area-based SES index**	4945					3751					4387					3330				
Lower tertile		1					1					1					1			
Medium tertile		1.31	(1.05–1.65)	0.018	0.18		1.34	(1.03–1.74)	0.028	0.25		1.29	(1.02–1.64)	0.033	0.16		1.34	(1.02–1.77)	0.034	0.35
Upper tertile		1.19	(0.94–1.51)	0.145			1.19	(0.91–1.57)	0.206			1.21	(0.94–1.55)	0.134			1.17	(0.88–1.56)	0.286	
Lowest 20%		1					1					1.00					1			
Highest 20%		1.33	(0.98–1.81)	0.071			1.39	(0.97–2.00)	0.069			1.32	(0.96–1.82)	0.092			1.30	(0.89–1.89)	0.172	

*ALL indicates acute lymphoblastic leukemia; 95% CI, 95% confidence interval; *N*, number of observation in the conditional regression model; *P*_trend_, *P*-value for trend over the SES levels*.

*^a^Adjusted for maternal age at birth, paternal age at birth, nationality, language region, older children in household*.

When repeating the analyses with the second and third best links datasets, we found similar results (Tables S1 and S2 in Supplementary Material). In further analyses, we investigated whether the strength of the SES association varied by time period (census 1990 versus census 2000) or by age at diagnosis (0–4 years versus 5–15 years). We did these analyses separately for all leukemia cases and for cases with acute lymphoblastic leukemia only. No effect modification between these two factors and any of the socioeconomic status measures was found (Table 4).

**Table 4:** **Interaction of *P*-values^a^ when assessing effect modification between SES measures and census (period of diagnosis) and SES measures and age at diagnosis, for leukemia (and ALL) respectively**.

	Census		Age at diagnosis	
	Leukemia	ALL	Leukemia	ALL
SES	*P*	*P*	*P*	*P*
Education status of the mother	0.229	0.139	0.761	0.782
Education status of the father	0.101	0.287	0.458	0.478
Rooms per person	0.650	0.606	0.828	0.760
Living space (in m^2^)	0.402	0.537	0.173	0.422
Area-based SES index	0.111	0.065	0.207	0.675

*ALL indicates acute lymphoblastic leukemia; *P*, *P*-values for interaction*.

*^a^Models were adjusted for maternal age, paternal age, nationality, language region, number of older children in household in addition to effect modification parameters*.

## Discussion

### Summary

In this Swiss population-based matched case (*N* = 559) -control (*N* = 4433) study, we found no consistent evidence for an association between socioeconomic status and risk of childhood leukemia or ALL. The associations did not change substantially with the operational definition of SES (individual-based, household-based, or area-based SES), time (comparing the periods 1990–2000 and 2000–2006), or age at diagnosis. Our results show that the included measures of socioeconomic status did not act as considerable risk factors or strong confounders in the etiology of childhood leukemia.

### Comparison with other studies

The results on the association between SES and incidence of leukemia published in the last years remain inconsistent (17, 20, 32). Researchers who had found a potential association had usually reported it only from the most extreme SES groups (the highest or lowest 10–20%) (16, 17, 30, 31). Therefore, we analyzed our continuous exposure measures (living space, area-based SES index) in two ways: comparing tertiles and comparing the two most extreme quintiles. Both analyses did not result in a significant association. The trend for a higher risk in the least deprived group (most living space, highest area-based index) was far from being statistically significant. Any true and causal association would be too small to explain an appreciable proportion of leukemia cases in childhood. Similarly, any of the included SES indicators would unlikely act as a strong confounder in other childhood leukemia incidence studies. Last, we did not find evidence that the association might have changed over time, as suggested in a review by Poole et al. (19). Our results are in line with the findings presented in a large population-based study of the Haematological Malignancy Research Network, in which the researchers questioned the benefit of future etiological investigations that focus solely on socioeconomic factors (33).

### Strengths and limitations

The chosen study design allowed us to overcome most limitations of earlier studies on SES and incidence of childhood leukemia. First, the study was nationwide and could largely avoid case and control participation bias, as active participation for cases and controls was not required. The sample included all leukemia cases of the childhood cancer registry fulfilling the criteria for linkage with the census datasets. Controls were drawn from the census, which are virtually complete datasets of the Swiss population. Participation bias was a major issue in many preceding studies (19, 20), as illustrated by Smith and co-authors, who simulated effects of control and case participation bias in their analysis (34). Second, by obtaining the information on socioeconomic status for both cases and controls from the census, we avoided reporting bias. Third, due to the linkage design (the children had to be born before the census and diagnosed after the census), the information on socioeconomic status was always collected before the cancer diagnosis. We can therefore be sure that the exposure preceded the outcome (e.g., SES did not change as a consequence of the diagnosis, for instance, if one of the parents had stopped working in order to care for the child). Fourth, the availability of different operational definitions of socioeconomic status with data on individual-based information (education), household-based information (number of rooms per person, square meter of living space per person), and area-based SES index is an important strength of our study. This allowed assessing the robustness of the association between SES and leukemia, and analyzing potential differences between these measures as previously suggested (19, 30). Last, information on potential leukemia risk factors associated with SES (maternal and paternal age; nationality; language region; number of children in household) minimized the potential for confounding.

This study has its limitations. First, the probabilistic linkage might have incorrectly linked some of the childhood cancer cases to a census child creating measurement error in the information of socioeconomic status. We addressed this issue by repeating the analyses with different linkage probability datasets. The results did not materially change. Second, the assignment of father and mother status in the same household might be incorrect, since male and female adults living in the same household are not necessarily biological fathers or mothers of the child. However, if there was a true SES leukemia association, one might argue that the socioeconomic status of the child is determined by the householder(s) it lives with. Third, the householders represent a relatively affluent and homogeneous population (Swiss nationals, well educated, employed, male head of household). Fourth, to have SES information preceding the diagnosis of leukemia, we excluded by design all children with leukemia born after census 1990 and diagnosed before census 2000, which leads to an underrepresentation of younger leukemia cases. This selection could lead to biased results if the association of SES is non-zero but varies with age. However, we did not find strong evidence against a common effect over age groups when testing for effect modification by age. If we had, in contrary, included these children, we would possibly have introduced survivor bias, because survival might be linked to SES and cases dying before 2000 were not linkable and would have missing SES information. Additionally, case registration in the childhood cancer registry was not complete during the first years. Completeness of the SCCR, estimated by the proportion of patients that first came to the registries attention via death certificate notification, was 85% in 1985–1989, 90% from 1990 to 1994, and ≥95% since 1995 (data not shown). Fifth, as we could only use information on socioeconomic status available in the census, we could not include any measure reflecting directly income or wealth. Sixth, as our sample size, although nationwide, was reduced by the inclusion criteria for our linkage design, statistical power was limited. This reduces particularly the ability to find small non-zero effects and to assess effects in subgroups, such as age groups (e.g., 1- to 4-year olds) or in rare leukemia subtypes (e.g., acute myeloid leukemia). Seventh, we were only able to include information available in the SCCR or the SNC and did hence not dispose of information on lifestyle-related risk factors, such as exposure to infections (e.g., nursery care) or to environmental tobacco smoke. Eight, the transferability of our results to other countries might be restricted. Evidence on the association of childhood leukemia with SES has been inconsistent across nations (20). An explanation for these inconsistencies might be the difficulties in the measurement of SES, possibly to a varying degree across studies and nations. Furthermore, SES might actually not be causally associated with childhood leukemia but merely be a marker for a varying degree of exposure to a true risk factor, again possibly varying between studies and countries. In the latter situation, results from one country would not be transferable to other countries. A last limitation concerns the timing of the SES information in the life course of the child. For a child born in 1990, the 1990 census information reflects SES at birth. For a child born in 1984 and diagnosed with leukemia in 1992, the census 1990 reflects SES at age 6. By individually matching controls to cases by year of birth, we guaranteed that the variability of timing in the life course was the same for cases and controls. However, if socioeconomic status particularly matters during a specific age period, as suggested by Raaschou-Nielsen and co-authors (30), we might have diluted such an association.

### Implications of our results

Given the various analyses performed, we concluded that the results show no consistent evidence for an effect of the included socioeconomic status indicators and the risk of childhood leukemia. The strongest association found with maternal but not paternal education might imply the involvement of intrauterine or postnatal factors, such as smoking behavior, occupational exposures, or dietary habits of the mother. As families with a mother of higher education usually exhibit lower prevalence of indoor cigarette smoking, higher health consciousness including attention to dietary habits, and lower occupational exposures to solvents, the observed association is unexpected (35–37). Future studies should take a life-course approach (38, 39) by assessing measures of SES at different developmental stages in order to distinguish the potential influence of SES during the separate stages of intrauterine development, infancy, and early childhood. SES *per se* is not a direct cause of leukemia, but rather a proxy measure indicating unequal distribution of a number of environmental and familial factors, which could influence the likelihood of a child to develop leukemia. To understand the chain of causation, it will be inevitable to study the relation of specific SES indicators with potential leukemia risk factors in great detail, as the risk factors for childhood leukemia remain poorly understood (40–45). Some potential SES-associated risk factors, for instance, paternal and maternal age, and number of older and younger siblings, have directly been considered in this study. A further, much discussed potential cause of leukemia is early or delayed exposure to infectious diseases during pregnancy and early childhood, with an influence on the development of the immune system of the child (18). By adjusting the numbers of older siblings, we have partly accounted for this, but had no information on nursery care, another risk factor for exposure to infections. Similarly, unhealthy lifestyle and higher exposure to a number of environmental factors (e.g., environmental tobacco smoke, different household chemical compounds, or radiation) are socially patterned and usually more prevalent in the lower SES groups being associated with a higher leukemia incidence risk. However, with the exception of parental smoking (46), none of these factors have been consistently and strongly associated with leukemia risk in children.

In conclusion, this carefully designed study did not find consistent evidence for an association between different SES definitions (parental education, living condition, area-based SES) and incidence of childhood leukemia. The included SES indicators are unlikely to be strong risk factors or confounding factors for leukemia incidence in children, in Switzerland or comparable countries. Future studies should therefore carefully define socioeconomic indicators and critically interpret their role when investigating the etiology of childhood leukemia.

## Author Contributions

CK and MZ designed the study. AS and KS contributed and interpreted the data from the Swiss National Cohort. FGP and PB contributed data to the Swiss Childhood Cancer Registry and interpreted the data from the perspective of the Swiss Paediatric Oncology Group. NPH interpreted the data as an expert in cancer epidemiology. MA and MZ analyzed the data. MA, CK, and MZ wrote the first draft of the manuscript. All authors of this research paper have directly participated in the planning, execution, or analysis of the study and revised the manuscript.

## Conflict of Interest Statement

The authors declare that the research was conducted in the absence of any commercial or financial relationships that could be construed as a potential conflict of interest.

## Supplementary Material

The Supplementary Material for this article can be found online at http://journal.frontiersin.org/article/10.3389/fonc.2015.00139

## Footnotes

^1^www.childhoodcancerregistry.ch

^2^www.swissnationalcohort.ch

^3^www.bfs.admin.ch/bfs/portal/de/index/regionen/03/dos/gesellschaft_und_kultur/01.html
