# Supplementary material for: Socioeconomic Status and Childhood Leukemia Incidence in Switzerland
Source: Front Oncol. 2015 Jun 30;5:139. doi: 10.3389/fonc.2015.00139 (PMC4485172; doi:10.3389/fonc.2015.00139)
Supplement: Supplementary file 1 [file Table_1.DOC]

**Supplementary Table S1** Risk association of socioeconomic status with leukemia (and ALL^a^), by type of SES characteristic; based on the second best links

|  | **Univariable analysis** | | | | | | | | **Multivariable analysis^b^** | | | | | | | |
| --- | --- | --- | --- | --- | --- | --- | --- | --- | --- | --- | --- | --- | --- | --- | --- | --- |
|  | **Leukemia** | | | | **ALL^a^** | | | | **Leukemia** | | | | **ALL^a^** | | | |
| **SES** | **N** | **OR** | **(95%CI)** | **P** | **N** | **OR** | **(95%CI)** | **P** | **N** | **OR** | **(95%CI)** | **P** | **N** | **OR** | **(95%CI)** | **P** |
|  |  |  |  |  |  |  |  |  |  |  |  |  |  |  |  |  |
| **Education status of the mother** | **4891** |  |  |  | **3704** |  |  |  | **4362** |  |  |  | **3340** |  |  |  |
| compulsory education |  | 1 |  |  |  | 1 |  |  |  | 1 |  |  |  | 1 |  |  |
| secondary education |  | 1.27 | (1.03-1.55) | 0.023 |  | 1.27 | (1.00-1.60) | 0.047 |  | 1.31 | (1.03-1.66) | 0.026 |  | 1.28 | (0.98-1.68) | 0.069 |
| tertiary education |  | 1.12 | (0.81-1.53) | 0.497 |  | 1.13 | (0.79-1.63) | 0.506 |  | 1.22 | (0.86-1.72) | 0.261 |  | 1.13 | (0.76-1.68) | 0.549 |
|  |  |  |  |  |  |  |  |  |  |  |  |  |  |  |  |  |
| **Education status of the father** | **4429** |  |  |  | **3393** |  |  |  | **4362** |  |  |  | **3340** |  |  |  |
| compulsory education |  | 1 |  |  |  | 1 |  |  |  | 1 |  |  |  | 1 |  |  |
| secondary education |  | 0.91 | (0.72-1.14) | 0.417 |  | 0.99 | (0.76-1.28) | 0.931 |  | 0.83 | (0.64-1.08) | 0.162 |  | 0.89 | (0.66-1.20) | 0.457 |
| tertiary education |  | 0.78 | (0.61-1.01) | 0.063 |  | 0.80 | (0.60-1.08) | 0.142 |  | 0.70 | (0.52-0.93) | 0.015 |  | 0.68 | (0.49-0.95) | 0.025 |
|  |  |  |  |  |  |  |  |  |  |  |  |  |  |  |  |  |
| **Rooms per person** | **4881** |  |  |  | **3686** |  |  |  | **4296** |  |  |  | **3279** |  |  |  |
| < 1 room/person |  | 1 |  |  |  | 1 |  |  |  | 1 |  |  |  | 1 |  |  |
| 1-1.25 room/person |  | 0.96 | (0.77-1.20) | 0.726 |  | 0.99 | (0.76-1.27) | 0.920 |  | 0.97 | (0.76-1.23) | 0.788 |  | 0.97 | (0.74-1.27) | 0.807 |
| > 1.25 room/person |  | 1.00 | (0.80-1.25) | 0.985 |  | 0.97 | (0.75-1.26) | 0.829 |  | 0.98 | (0.75-1.27) | 0.867 |  | 0.91 | (0.67-1.23) | 0.536 |
|  |  |  |  |  |  |  |  |  |  |  |  |  |  |  |  |  |
| **Living space (in m2)** | **3613** |  |  |  | **2733** |  |  |  | **3196** |  |  |  | **2448** |  |  |  |
| lower tertile |  | 1 |  |  |  | 1 |  |  |  | 1 |  |  |  | 1 |  |  |
| medium tertile |  | 0.93 | (0.74-1.18) | 0.570 |  | 0.91 | (0.70-1.19) | 0.502 |  | 0.99 | (0.76-1.28) | 0.926 |  | 0.95 | (0.71-1.27) | 0.712 |
| upper tertile |  | 1.09 | (0.86-1.38) | 0.491 |  | 1.04 | (0.79-1.37) | 0.766 |  | 1.08 | (0.82-1.41) | 0.583 |  | 1.00 | (0.73-1.35) | 0.979 |
|  |  |  |  |  |  |  |  |  |  |  |  |  |  |  |  |  |
| lowest 20 % |  | 1 |  |  |  | 1 |  |  |  | 1.00 |  |  |  | 1 |  |  |
| highest 20 % |  | 1.04 | (0.77-1.41) | 0.796 |  | 1.03 | (0.73-1.46) | 0.861 |  | 1.06 | (0.75-1.51) | 0.741 |  | 0.96 | (0.65-1.44) | 0.859 |
|  |  |  |  |  |  |  |  |  |  |  |  |  |  |  |  |  |
| **Area-based SES index** | **4964** |  |  |  | **3761** |  |  |  | **4362** |  |  |  | **3340** |  |  |  |
| lower tertile |  | 1 |  |  |  | 1 |  |  |  | 1 |  |  |  | 1 |  |  |
| medium tertile |  | 1.10 | (0.88-1.38) | 0.421 |  | 1.04 | (0.80-1.35) | 0.782 |  | 1.02 | (0.80-1.29) | 0.873 |  | 1.00 | (0.76-1.31) | 0.983 |
| upper tertile |  | 1.08 | (0.86-1.37) | 0.498 |  | 1.11 | (0.85-1.45) | 0.464 |  | 1.07 | (0.84-1.37) | 0.573 |  | 1.07 | (0.81-1.41) | 0.657 |
|  |  |  |  |  |  |  |  |  |  |  |  |  |  |  |  |  |
| lowest 20 % |  | 1 |  |  |  | 1 |  |  |  | 1.00 |  |  |  | 1 |  |  |
| highest 20 % |  | 1.05 | (0.78-1.42) | 0.750 |  | 1.11 | (0.79-1.57) | 0.549 |  | 1.03 | (0.75-1.42) | 0.838 |  | 1.03 | (0.72-1.49) | 0.860 |
|  |  |  |  |  |  |  |  |  |  |  |  |  |  |  |  |  |

^a^ ALL = acute lymphoblastic leukemia

^b^ Adjusted for: maternal age at birth, paternal age at birth, nationality, language region, older children in household

N = Number of observation in the conditional regression model
