# Supplementary material for: Socioeconomic Status and Childhood Leukemia Incidence in Switzerland
Source: Front Oncol. 2015 Jun 30;5:139. doi: 10.3389/fonc.2015.00139 (PMC4485172; doi:10.3389/fonc.2015.00139)
Supplement: Supplementary file 2 [file Table_2.DOC]

**Supplementary Table S2** Risk association of socio-economic status with Leukemia (and ALL^a^), by type of SES characteristic; based on the third best links

|  | **Univariable analysis** | | | | | | | | **Multivariable analysis^b^** | | | | | | | |
| --- | --- | --- | --- | --- | --- | --- | --- | --- | --- | --- | --- | --- | --- | --- | --- | --- |
|  | **Leukemia** | | | | **ALL^a^** | | | | **Leukemia** | | | | **ALL^a^** | | | |
| **SES** | **N** | **OR** | **(95%CI)** | **P** | **N** | **OR** | **(95%CI)** | **P** | **N** | **OR** | **(95%CI)** | **P** | **N** | **OR** | **(95%CI)** | **P** |
|  |  |  |  |  |  |  |  |  |  |  |  |  |  |  |  |  |
| **Education status of the mother** | **4908** |  |  |  | **3721** |  |  |  | **4415** |  |  |  | **3366** |  |  |  |
| compulsory education |  | 1 |  |  |  | 1 |  |  |  | 1 |  |  |  | 1 |  |  |
| secondary education |  | 1.21 | (0.99-1.49) | 0.062 |  | 1.23 | (0.97-1.55) | 0.083 |  | 1.25 | (0.99-1.59) | 0.059 |  | 1.23 | (0.94-1.61) | 0.136 |
| tertiary education |  | 1.25 | (0.93-1.70) | 0.144 |  | 1.34 | (0.95-1.89) | 0.096 |  | 1.34 | (0.96-1.86) | 0.085 |  | 1.32 | (0.91-1.93) | 0.143 |
|  |  |  |  |  |  |  |  |  |  |  |  |  |  |  |  |  |
| **Education status of the father** | **4481** |  |  |  | **3410** |  |  |  | **4415** |  |  |  | **3366** |  |  |  |
| compulsory education |  | 1 |  |  |  | 1 |  |  |  | 1 |  |  |  | 1 |  |  |
| secondary education |  | 1.12 | (0.89-1.42) | 0.342 |  | 1.22 | (0.93-1.60) | 0.159 |  | 1.06 | (0.81-1.39) | 0.655 |  | 1.13 | (0.83-1.55) | 0.428 |
| tertiary education |  | 0.93 | (0.71-1.21) | 0.588 |  | 0.99 | (0.73-1.34) | 0.934 |  | 0.85 | (0.63-1.14) | 0.266 |  | 0.87 | (0.62-1.22) | 0.411 |
|  |  |  |  |  |  |  |  |  |  |  |  |  |  |  |  |  |
| **Rooms per person** | **4899** |  |  |  | **3695** |  |  |  | **4351** |  |  |  | **3307** |  |  |  |
| < 1 room/person |  | 1 |  |  |  | 1 |  |  |  | 1 |  |  |  | 1 |  |  |
| 1-1.25 room/person |  | 0.96 | (0.77-1.20) | 0.701 |  | 1.00 | (0.78-1.29) | 0.989 |  | 0.99 | (0.78-1.26) | 0.918 |  | 1.03 | (0.78-1.35) | 0.850 |
| > 1.25 room/person |  | 1.00 | (0.80-1.25) | 0.982 |  | 0.94 | (0.73-1.22) | 0.657 |  | 1.00 | (0.77-1.31) | 0.973 |  | 0.94 | (0.69-1.27) | 0.686 |
|  |  |  |  |  |  |  |  |  |  |  |  |  |  |  |  |  |
| **Living space (in m2)** | **3794** |  |  |  | **2849** |  |  |  | **3383** |  |  |  | **2569** |  |  |  |
| lower tertile |  | 1 |  |  |  | 1 |  |  |  | 1 |  |  |  | 1 |  |  |
| medium tertile |  | 0.98 | (0.78-1.23) | 0.846 |  | 0.91 | (0.70-1.18) | 0.456 |  | 1.04 | (0.81-1.33) | 0.777 |  | 0.95 | (0.72-1.26) | 0.739 |
| upper tertile |  | 1.07 | (0.84-1.35) | 0.589 |  | 0.94 | (0.72-1.23) | 0.663 |  | 1.05 | (0.81-1.37) | 0.717 |  | 0.92 | (0.68-1.24) | 0.568 |
|  |  |  |  |  |  |  |  |  |  |  |  |  |  |  |  |  |
| lowest 20 % |  | 1 |  |  |  | 1 |  |  |  | 1.00 |  |  |  | 1 |  |  |
| highest 20 % |  | 1.05 | (0.78-1.41) | 0.765 |  | 0.96 | (0.68-1.35) | 0.821 |  | 1.05 | (0.74-1.49) | 0.768 |  | 0.96 | (0.65-1.41) | 0.820 |
|  |  |  |  |  |  |  |  |  |  |  |  |  |  |  |  |  |
| **Area-based SES index** | **4981** |  |  |  | **3769** |  |  |  | **4415** |  |  |  | **3366** |  |  |  |
| lower tertile |  | 1 |  |  |  | 1 |  |  |  | 1 |  |  |  | 1 |  |  |
| medium tertile |  | 1.05 | (0.84-1.31) | 0.683 |  | 1.07 | (0.83-1.39) | 0.594 |  | 1.00 | (0.79-1.26) | 0.993 |  | 1.02 | (0.78-1.33) | 0.889 |
| upper tertile |  | 0.96 | (0.76-1.22) | 0.758 |  | 0.98 | (0.74-1.28) | 0.873 |  | 0.95 | (0.74-1.21) | 0.662 |  | 0.92 | (0.69-1.22) | 0.555 |
|  |  |  |  |  |  |  |  |  |  |  |  |  |  |  |  |  |
| lowest 20 % |  | 1 |  |  |  | 1 |  |  |  | 1.00 |  |  |  | 1 |  |  |
| highest 20 % |  | 1.00 | (0.74-1.35) | 0.990 |  | 1.01 | (0.71-1.44) | 0.956 |  | 0.98 | (0.71-1.35) | 0.913 |  | 0.92 | (0.63-1.33) | 0.645 |
|  |  |  |  |  |  |  |  |  |  |  |  |  |  |  |  |  |

^a^ ALL = acute lymphoblastic leukemia

^b^ Adjusted for: maternal age at birth, paternal age at birth, nationality, language region, older children in household

N = Number of observation in the conditional regression model
