# Supplementary material for: Socioeconomic Status and Childhood Leukemia Incidence in Switzerland
Source: Front Oncol. 2015 Jun 30;5:139. doi: 10.3389/fonc.2015.00139 (PMC4485172; doi:10.3389/fonc.2015.00139)
Supplement: Supplementary file 3 [file Data_Sheet_1.DOCX]

**Supplementary Material**

Socioeconomic status and childhood leukemia incidence in Switzerland

Martin Adam^1,2,3^*^#^, Claudia Kuehni^3#^, Adrian Spoerri^3^, Kurt Schmidlin^3^, Fabienne Gumy-Pause^4^, Pierluigi Brazzola^5^, Nicole Probst-Hensch^1,2^, Marcel Zwahlen^3^ for the Swiss Paediatric Oncology Group (SPOG) and the Swiss National Cohort Study Group (SNC)

^#^ both authors contributed equally

^1^ Swiss Tropical and Public Health Institute, 4002 Basel, Switzerland

^2^ University of Basel, 4001 Basel, Switzerland

^3^ Institute of Social and Preventive Medicine, University of Bern, 3012 Bern, Switzerland

^4^ Haematology/Oncology Unit, Department of Paediatrics, University Hospital of Geneva, 1205 Geneva, Switzerland

^5^ Department of Paediatrics, Ospedale San Giovanni, 6500 Bellinzona, Switzerland

*** Correspondence:**

Dr. Martin Adam

Swiss Tropical and Public Health Institute,

Socinstrasse 57, 4002 Basel, Switzerland;

E-mail: martin.adam@unibas.ch;

Phone: +41 61 284 83 96, Fax: +41 61 284 81 05

**SUPPLEMENTARY METHODS S1**

**Linkage with the Swiss National Cohort**

Data preparation in the SCCR included several adaptations of the SCCR dataset to codes and norms used in the census dataset of the SNC. All postcodes for address at diagnosis and current address were mapped into community codes, and cantonal codes according to code lists issued by the Swiss Federal Statistical Office (SFSO). A probabilistic record linkage method was used to link the record in the census dataset to case records because no unique numeric personal identifier is available in Switzerland. We linked records from the 1990 census to the “census 1990 case subset” and records from the 2000 census to the “census 2000 case subset” using the Generalized Record Linkage System (GRLS) software package developed by Statistics Canada ([1](#_ENREF_1)). For the record linkage information available both in the census records and in the SCCR was used: sex, date of birth, place of residence. Probabilistic record linkage provides probability weights that a pair of records from different datasets relates to the same person. The weights for each linkage attribute are summed up to build a total linkage probability weight for each possible link. Based on this weight, by threshold the possible links are categorized as a) definite match, b) probable match or c.) rejected match. Since in some instances several potential links with a different linkage probability weight per record were found, we prepared three different data sets for sensitivity analyses: i) best links data set (including all definite links and of the non-definitive links the one with the highest weight), ii) second best links data set (including all definite links and of the non-definitive links the one with the second highest weight) and iii) third best links data set (including all definite links and of the non-definitive links the one with the third highest weight).

**REFERENCES**

1. Fair M. Generalized Record Linkage System - Statisticis Canada's record linkage software. *Austrian Journal of Statisticis* (2004) **33**(37):53. PubMed PMID: 4944.
